# Combinatorial drug screening and molecular profiling reveal diverse mechanisms of intrinsic and adaptive resistance to BRAF inhibition in V600E BRAF mutant melanomas

**DOI:** 10.18632/oncotarget.6548

**Published:** 2015-12-10

**Authors:** Devin G. Roller, Brian Capaldo, Stefan Bekiranov, Aaron J. Mackey, Mark R. Conaway, Emanuel F. Petricoin, Daniel Gioeli, Michael J. Weber

**Affiliations:** ^1^ Department of Microbiology, Immunology, and Cancer Biology, University of Virginia, Charlottesville, VA, 22908 USA; ^2^ Department of Biochemistry and Molecular Genetics, University of Virginia, Charlottesville, VA, 22908 USA; ^3^ Department of Public Health Sciences, University of Virginia, Charlottesville, VA, 22908 USA; ^4^ Center for Applied Proteomics and Molecular Medicine, School of Systems Biology, College of Science, George Mason University, Manassas, VA 20110, USA

**Keywords:** melanoma, therapeutic resistance, cell signaling, BRAF, MAP Kinase

## Abstract

Over half of BRAFV600E melanomas display intrinsic resistance to BRAF inhibitors, in part due to adaptive signaling responses. In this communication we ask whether BRAFV600E melanomas share common adaptive responses to BRAF inhibition that can provide clinically relevant targets for drug combinations. We screened a panel of 12 treatment-naïve BRAFV600E melanoma cell lines with MAP Kinase pathway inhibitors in pairwise combination with 58 signaling inhibitors, assaying for synergistic cytotoxicity. We found enormous diversity in the drug combinations that showed synergy, with no two cell lines having an identical profile. Although the 6 lines most resistant to BRAF inhibition showed synergistic benefit from combination with lapatinib, the signaling mechanisms by which this combination generated synergistic cytotoxicity differed between the cell lines. We conclude that adaptive responses to inhibition of the primary oncogenic driver (BRAFV600E) are determined not only by the primary oncogenic driver but also by diverse secondary genetic and epigenetic changes (“back-seat drivers”) and hence optimal drug combinations will be variable. Because upregulation of receptor tyrosine kinases is a major source of drug resistance arising from diverse adaptive responses, we propose that inhibitors of these receptors may have substantial clinical utility in combination with inhibitors of the MAP Kinase pathway.

## INTRODUCTION

BRAF inhibitors such as vemurafenib and dabrafenib have generated remarkable responses in the approximately 50% of melanomas containing activating mutations in BRAF [See reference [1] for a comprehensive review]. However, only 50–60% of patients demonstrate therapeutic responses to BRAF inhibitors by RECIST criteria, and only 10% demonstrate a complete response. Moreover, the responses generally are not durable, with aggressive disease typically recurring within 6 months [2]. When disease recurs it often appears at the same sites as the original tumors [3], indicating that even in cases where responses appear to be robust, many tumors are intrinsically resistant to treatment because of their ability to rapidly adapt to target inhibition. Even though combining a MEK inhibitor such as trametinib along with a BRAF inhibitor can improve the response, only half of patients show a “complete” response and the majority of patients show disease progression after one year [4–8].

Analysis of samples from patients with recurrent disease has provided considerable insight into the mechanisms by which BRAF mutant melanomas ultimately achieve resistance to BRAF inhibitors [6, 8–20]. These almost always involve reactivation of the MAP Kinase pathway, although PI3Kinase, STATs, HIPPO, beta-catenin, BH3 proteins, autophagy and translational regulation have also been flagged [21–40]. Tumors that have been selected for resistance can be expected to display diverse resistance mechanisms, since anything that promotes cell growth can provide a selective advantage for the tumor. Indeed, multiple resistance mechanisms appear even between different metastases or different regions within the same tumor [16, 20, 41–43].

Less is known about the rapid homeostatic adaptations that occur within the first few hours or days following the initiation of treatment and that allow the cancer cells to survive initial inhibition of BRAF. A number of investigators [18, 44–47] have called attention to these homeostatic adaptations as an important component of intrinsic resistance or early relapse, and pointed out how they can set the stage for selection of genetic and epigenetic variants in which the MAP Kinase pathway is reactivated. Thus, there is a need to identify and analyze the adaptations that can be deployed rapidly by cancer cells and that enable survival and the resumption of proliferation in spite of inhibition of mutant BRAF. It seems likely that identification and analysis of mechanisms of adaptive resistance to BRAF inhibition could guide the development of additional combination therapies that would provide more complete responses, and by anticipating mechanisms of acquired resistance, would lead to more durable responses as well.

Analysis of transcriptional and proteomic changes following blockade of MAP Kinase signaling has revealed a stunning complexity in adaptive responses [8, 20, 41, 45–61] and it has been difficult to determine which of the numerous components of the “adaptome” would be most appropriate for therapeutic co-targeting. Therefore, we [61, 62] as well as Held et al. [56] have taken an empirical, chemical genetic approach to identify actionable adaptive responses. Both groups performed chemical genetic screens with diverse drug combinations to probe the melanoma cell signaling network for novel functional interactions and identify drug combinations effective on either mutant RAF, RAS or wild-type melanomas. Surprisingly, no single drug combination or subset of drug combinations was found to be synergistic in either all of the RAF or all of the RAS mutant melanoma lines. This suggested that the underlying signaling network of each melanoma line was different, even when the primary driver (RAF or RAS) was the same.

In the current communication we focused on identifying drug combinations that might be clinically useful and mechanistically informative. We conducted a targeted combinatorial chemical genetic screen using as primary drugs either the vemurafenib analog PLX4720 or two other inhibitors of the MAP Kinase pathway in two-way combinations with 58 drugs or clinically relevant tool compounds in 12 BRAFV600E melanoma cell lines. We found that half the lines showed synergistic benefit by combining lapatinib or masitinib with PLX4720. Importantly, the lines that showed benefit from this combination were the lines least sensitive intrinsically to BRAF inhibition, indicating the importance of Receptor Tyrosine Kinase (RTK) signaling in vemurafenib resistance, as shown previously [13, 56, 63–75]. Nevertheless, the overall pattern of effective drug combinations was different for each cell line, indicating that the “wiring” of the signaling network and mechanisms of adaptive resistance differed for each line even though all were driven by BRAFV600E and 6 of the 12 showed enhanced cytotoxicity from an RTK inhibitor. Protein pathway phosphorylation/activation mapping via reverse phase protein arrays (RPPA) and gene expression analysis confirmed that, even when cells were sensitive to the combination of PLX4720 and lapatinib, the adaptive changes in intracellular signaling in response to BRAF inhibition differed and the mechanism(s) by which lapatinib or masitinib were synergistically cytotoxic differed. We propose that intrinsic and adaptive resistance to BRAF inhibition in BRAFV600E melanomas occurs by multiple mechanisms that differ substantially, dependent on the broader genetic and epigenetic landscape of the cancer cells that shape the underlying architecture of cell signaling networks. Because Receptor Tyrosine Kinases (RTKs) can activate multiple resistance pathways, and upregulation of RTKs is a convergent source of drug resistance in many cell lines, inhibitors of these receptors can play an important role in drug combinations in a variety of genetic backgrounds and may be clinically useful in combinations with inhibitors of the MAP Kinase pathway.

## RESULTS

### Combinatorial drug screening reveals diverse patterns of cytotoxic synergy between MAP Kinase pathway inhibitors and other targeted agents

**Figure 1:**
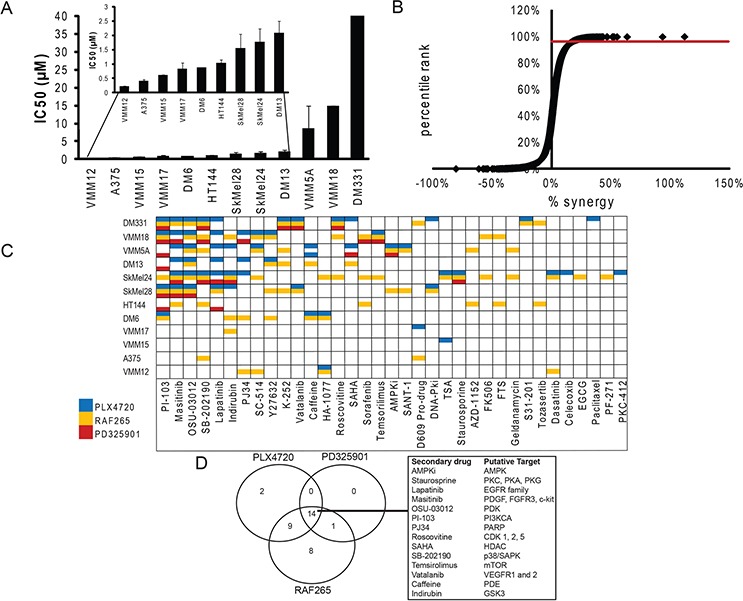
Synthetic lethal screen identifies synergistic drug combinations **A.** Cells were treated with varying doses of PLX4720 for 3 days to determine an IC50 for each cell line. Cytotoxicity was read out using alamarBlue (*n* = 3). **B.** A synthetic lethal screen was performed by combining 58 secondary drugs with varying concentrations of the vemurafenib-analog, PLX4720, pan-RAF inhibitor, RAF265, or MEK inhibitor, PD325901 on 12 BRAF mutant melanoma cell lines. Percent cytotoxicity was measured with an alamarBlue assay, and percent synergy assessed by the Bliss independence method [76]. Cytotoxicity was normalized to the vehicle control treated samples for each cell line. Each data point on the curve represents the difference between the observed cytotoxicity and the predicted additive cytotoxicity based on the Bliss model (termed “percent synergy”). A cutoff was drawn at *p*-value of 0.05 which corresponds to the 98^th^ percentile and 26% synergy. **C.** Drug combinations that produced cytotoxicity greater than 26% over Bliss predicted were identified and color coded according to the primary drug (PLX4720 [blue bars], RAF265 [yellow bars] or PD325901 [red bars]). Combinations that did not display synergy in any line are not shown. **D.** Venn diagram shows overlap of synergistic cytotoxicity of secondary drug with Raf and MEK inhibitors.

We assembled a library of 58 small molecule cell signaling inhibitors, focused on targeted drugs or related tool compounds, and containing not only kinase inhibitors, but also inhibitors of various other enzymatic processes (Supplementary Table S1). These were tested for synergistic cytotoxicity in 12 treatment-naïve BRAFV600E cell lines in combination with one of three inhibitors of the MAP Kinase pathway: PLX4720, a vemurafenib analog and inhibitor of activated RAF; RAF265, a less-specific RAF inhibitor; or PD325901, a selective allosteric MEK inhibitor (Figure 1). We scored for synergy for two reasons. First, synergy is considered a marker of functional interactions between the targets, thus suggesting that the benefit of drug combination was due to inhibition of an adaptive response. Second, there is a possibility that the synergy would be maintained *in vivo*, thus enabling a broader therapeutic window. We scored synergy using the Bliss model of independence [76, 77] in part because it quantifies interactions even where one drug does not display cytotoxic effects as a single agent, a common occurrence with targeted therapies.

Figure 1 Panel A demonstrates the range of variability of intrinsic sensitivity in our cell line panel to PLX4720 as a single agent: from VMM12 and A375 which are highly sensitive, to DM331 which is almost entirely resistant *in vitro*. Panel B displays the degrees of synergistic or antagonistic interactions between the drug pairs, and identifies the 26% synergy (98^th^ percentile) cut-off that was used to generate Panel C, where the synergistic drug pairs are displayed. Note that each of the three MAP Kinase pathway inhibitors is synergistic with a distinct pattern of combination drugs, demonstrating that the specificity of the primary drug (e.g. PLX4720 vs RAF265) and the identity of its target (RAF vs MEK) play a role in establishing the optimum combination of drugs. This is displayed in Panel D, which shows that two of the secondary drugs demonstrated synergy only with PLX4720, and nine showed synergy with PLX4720 and RAF265 but not with the MEK inhibitor. No drugs were synergistic only with the MEK inhibitor, consistent with the notion that effectiveness of the RAF inhibitors depends at least in part on their inhibition of MEK activity. Also note that the frequency of synergistic “hits” was inversely proportional to the selectivity of the primary drug, indicating the potential utility of multi-target agents even in drug combinations. Importantly, no two cell lines showed the same pattern of sensitivity to drug combinations. This further confirms previous suggestions [56, 62] that the genetic landscape of each individual cell line is determinative for the ability of a drug combination's effectiveness, regardless of the cell lineage and primary driver, in this case BRAFV600E melanoma.

**Figure 2:**
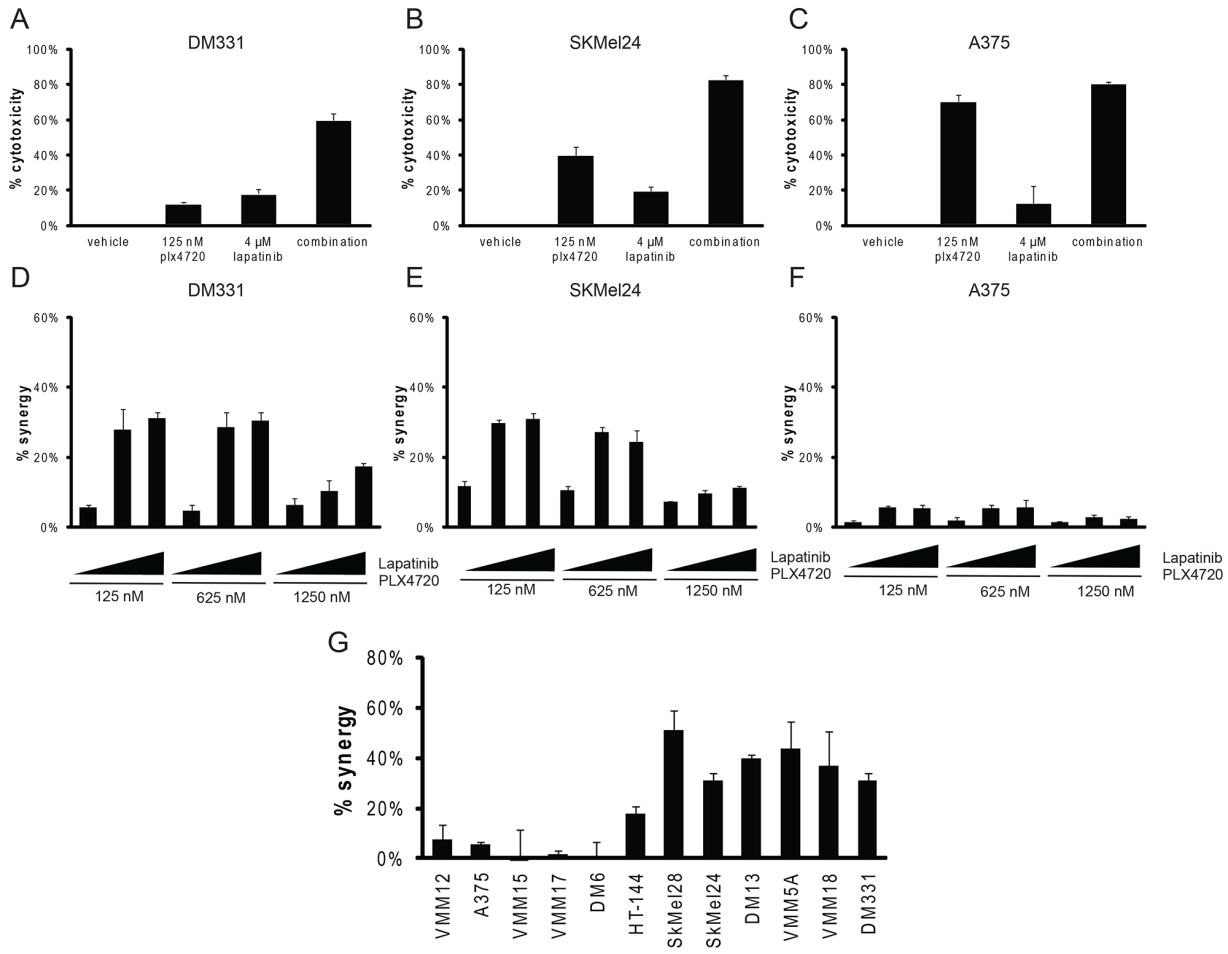
The combination of PLX4720 and lapatinib is synergistic *in vitro* **A, B, C.** B-raf mutant cells DM331, SkMel24 or A375 were treated with vehicle control, 125 nM plx4720, 4 μM lapatinib, or concurrent treatment of 125 nM plx4720 and 4 μM lapatinib for 3 days. Metabolic activity was read out using alamarBlue (*n* = 3). **D, E, F.** Dose dependent synergistic benefit was determined in cells concurrently treated with PLX4720 (125 nM, 625 nM, or 1250 nM) and lapatinib (1 μM, 2 μM, or 4 μM) for 3 days. AlamarBlue was used to read out metabolic activity. Percent synergy is displayed for each dose combination (*n* = 3). **G.** *BRAF* mutant cells: VMM12, A375, VMM15, VMM17, DM6, HT144, SkMel28, SKMel24, DM13, VMM5A, VMM18 and DM331 were treated with combinations of plx4720 and lapatinib for 3 days. AlamarBlue was used to read out metabolic activity. The average predicted Bliss value as plotted against the average actual cytotoxicity for each cell line (*n* = 3). Compare synergistic response to PLX4720 resistance shown in Figure 1A).

Within this complexity, there is a striking concordance: half of the cell lines showed synergy between PLX4720 and lapatinib or masitinib (Figure 1C), suggesting an important role for Receptor Tyrosine Kinase activity in intrinsic resistance to BRAFV600E inhibition, consistent with previous findings from others [13, 54, 56, 63, 64, 66–74, 78–85]. The data in Figure 2A–2F demonstrate that the synergistic interactions between lapatinib and PLX4720 are displayed over a wide range of concentrations, and that the combination can generate almost complete cytotoxicity in resistant cell lines at concentrations of individual drugs that have incomplete effects as single agents. By contrast, the PLX4720 sensitive line A375 showed little benefit and no significant synergistic effect from the addition of lapatinib, even though the experiments utilized at least one dose below the IC50. The effectiveness of lapatinib was not consistently correlated with the expression level of the EGFR or other members of the HER family of Receptor Tyrosine Kinases (Supplementary Figure S1), although DM331, the most resistant line, expressed the highest levels of EGFR and we have shown that it expresses neuregulin, and is the only line in this panel to do so [61]. Figure 2G demonstrates that the response to the PLX4720-lapatinib combination is bimodal with respect to synergy. Importantly, the lines that showed the greatest intrinsic resistance to PLX4720 as a single agent displayed synergistic benefit from the addition of lapatinib (Compare Figure 2G to Figure 1B). By contrast, the lines most sensitive to PLX4720 did not display synergistic cytotoxicity from lapatinib addition even when PLX4720 was used at concentrations that caused only modest (less than 50%) growth inhibition.

### Synergistic benefit from combining PLX4720 with lapatinib *in vivo*

**Figure 3:**
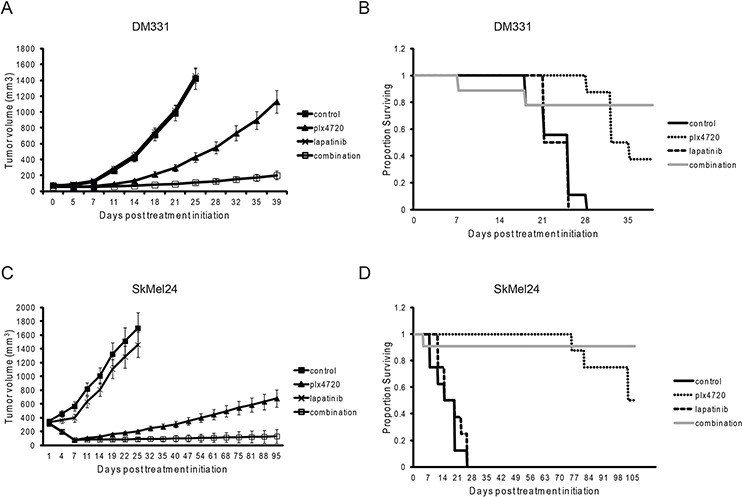
The combination of PLX4720 and lapatinib reduces DM331 and SKMEL24 xenograft tumor growth and extends mouse survival **A.** Growth of DM331 cells as xenografts in Hsd:Athymic Nude-Foxn1nu mice treated with lapatinib (200 mg/kg by oral gavage QID), rodent diet with 417 mg/kg plx4720 or the combination. Drug treatment commenced when DM331 tumors were 50–60 mm^3^. Tumor volume and standard error of the mean are shown (*n* = 8 in lapatinib and plx4720 groups, *n* = 9 in control and combination groups). **B.** Kaplan-Meier survival curve of DM331 xenograft following lapatinib, plx4720, or combination treatment of lapatinib and plx4720. **C.** Growth of SkMel24 cells as xenografts established and treated as above. Drug treatment commenced when SKMel24 tumors were 200–300 mm^3^. Tumor volume and standard error of the mean are shown (*n* = 8 per group). **D.** Kaplan-Meier survival curve of SkMel24 xenografts following lapatinib, plx4720, or combination treatment.

To determine whether synergy observed *in vitro* could be seen *in vivo*, PLX4720-resistant DM331 cells and partially resistant SKMEL24 cells were implanted subcutaneously in immunodeficient mice, PLX4720 was administered with or without lapatinib, and tumor growth was monitored. DM331 tumors were partially sensitive to PLX4720 *in vivo* even though they were almost entirely resistant in cell culture. We do not know whether this is due to reprogramming of the melanoma signaling networks *in vivo*, an effect on the tumor microenvironment or pharmacokinetic issues. Addition of lapatinib provided strong therapeutic benefit (Figure 3). Qualitatively similar results were obtained with SKMEL24 xenografts, showing improved tumor control with the drug combinations. The mice tolerated the combination well with all mice maintaining weight throughout the experiment (data not shown).

### BRAF inhibition triggers diverse adaptive responses in cell signaling

**Figure 4:**
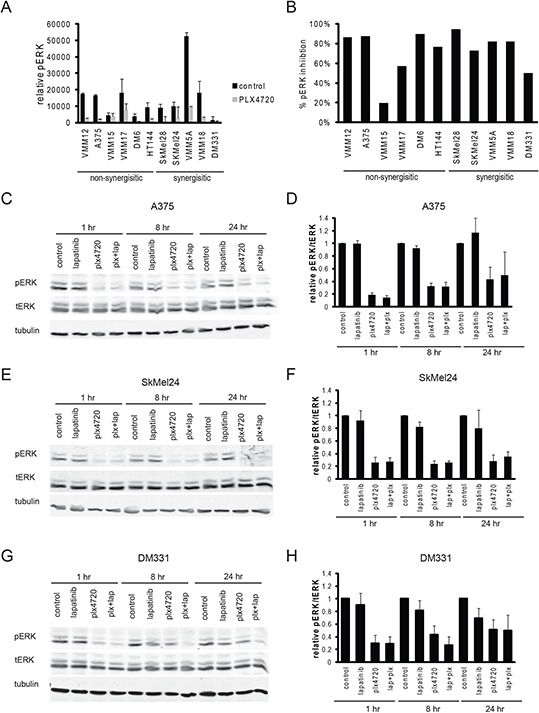
Inhibition of MAP Kinase occurs in sensitive and resistant cell lines **A.** Relative pERK levels were determined by Reverse Phase Protein Array of cells after treatment with vehicle control (black bars) or 8 hours of 125nM plx4720. (*n* = 3 per cell line) **B.** The percent pERK inhibition was calculated for each cell line. **C-H.** Cells were treated with vehicle control, 125nM plx470, 2 μM lapatinib, or the combination of plx4720 and lapatinib for 1, 8, or 24 hours. Total protein was isolated and immunoblot analysis was performed for pERK, tERK, and tubulin. A representative Western blot and qualification of the Western blot analysis (*n* = 3) is shown for (C, D) A375, (E, F) SkMel24, and (G, H) DM331.

Because resistance to BRAF inhibitors in melanoma patients is almost always due to reactivation of the MAP Kinase pathway [6, 11, 13, 15, 18, 20, 44, 60, 70, 84, 86–96] we expected that lapatinib would reinforce the effectiveness of PLX4720 on MAP Kinase pathway inhibition. However, western blots of phospho-ERK did not confirm this expectation (Figure 4): during the 72 hour period where growth inhibition was measured, comparable inhibition of ERK phosphorylation by PLX4720 was observed in sensitive and resistant lines at concentrations of PLX4720 where synergy was apparent, and lapatinib addition had little effect on this (although a modest effect on rebound of ERK phosphorylation in DM331 was observed in some experiments).

**Figure 5:**
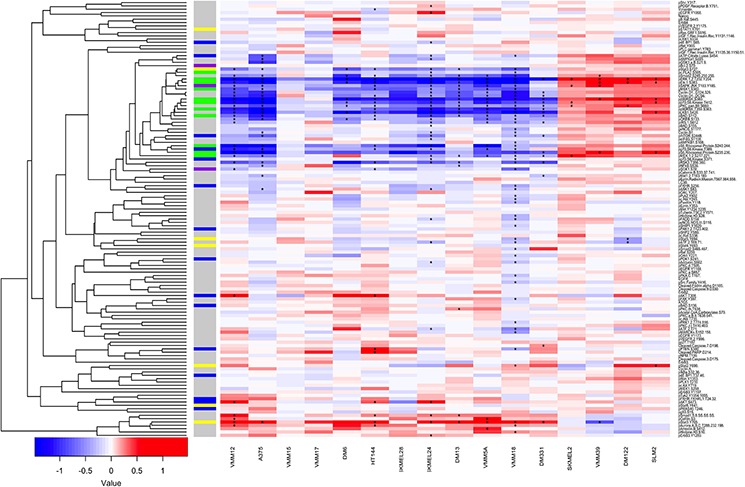
Reverse Phase Protein Arrays show diverse responses to PLX4720 in a panel of 16 melanoma cell lines 12 BRAFV600E melanoma cell lines and 4 BRAFwt lines were treated for 8 hrs with 125 μM PLX4720. Cells were lysed as described and analyzed by Reverse Phase Protein Arrays. Differentially abundant or differentially phosphorylated epitopes were identified using a moderated *t*-test, and epitopes with an FDR of better than 1% are marked with an asterisk (“*”). Unsupervised hierarchical clustering of log2 fold changes for epitopes (y-axis) was performed using correlation distance and average linkage. Cell lines are ordered by sensitivity to PLX4720 (IC50). Pathway membership of epitopes is denoted along the y-axis by the presence of colored boxes for the MAPK pathway (Green), STAT (yellow), PI3K pathway (Blue), stress pathways (Purple).

We employed RPPA to map the basal activation state and adaptive responses to BRAF inhibition on a broader range of signaling pathway proteins in our panel of 12 BRAFV600E melanomas as well as 4 BRAFwt melanomas (Figure 5 and Supplementary Figures 2, 3 and 5). In the basal state, phosphosites representative of the MAPK, PI3K JNK or STAT pathways did not correlate uniformly with sensitivity to PLX4720 or responsiveness to lapatinib. However, there was a trend for higher expression of pAKT and some of its substrates in the 8 lines most resistant to PLX4720, compared to 3 of the 4 most sensitive lines (Supplementary Figure S2). Similarly, by comparing protein and phosphoprotein levels from RPPA data between the three most resistant (VMM5A, VMM18 and DM331) and three most sensitive (VMM12, A375 and VMM15) cell lines to PLX4720 treatment using a Wilcoxon Rank Sum test, we identified significantly higher levels of pAKT and pAKT substrates in the basal state of the resistant lines (data not shown). However, these trends in basal phosphorylation did not correlate with sensitivity to the combination of PLX4720 with either PI3K or PDK1 inhibitors (Figures 1 and 7).

We then performed RPPA analysis to determine whether there was congruence in adaptive responses to BRAF inhibition. Cultures of the 12 BRAFV600E and 4 BRAFwt cell lines were treated with PLX4720 for 8 hours, a time chosen based on work showing that feedback responses to MAP Kinase pathway inhibition become evident at 6–8 hours [44, 97]. The data (Figure 5) show that BRAFV600E inhibition causes inhibition of many protein phosphorylations in addition to those that are considered part of the MAP Kinase pathway. Conversely, in the BRAFwt melanoma lines, MAP Kinase pathway phosphorylations are “paradoxically” upregulated as expected [98] but also many other phosphorylations are upregulated, presumably reflecting the adaptive changes for these genotypes. Besides this broad dichotomy, the diversity of adaptive responses within the BRAFV600E melanomas is striking. Notably, the degree of inhibition of MAP Kinase pathway components was not related to drug sensitivity, and upregulation of PI3 Kinase-related epitopes occurred in only a few instances. Unsupervised cluster analysis of the changes in protein phosphorylation induced by treatment with PLX4720 as a single agent within the panel of 114 phospho-sites revealed that the adaptive responses (“kinome rewiring” [46]) differed substantially between the cell lines mirroring the diversity of synergistic drug responses shown in Figure 1C, and no consistent signature of adaptive responses could be extracted.

To determine whether lapatinib would consistently have a predominant effect on a subset of these adaptive responses, we treated cells with PLX4720, lapatinib or the combination for 1, 8 or 24 hours and analyzed the responses by RPPA (Supplementary Figure S3). We included PLX4720-sensitive A375 cells, and three lines that covered the span of resistance: SKMEL24, VMM5A and DM331, as well as two RAS-transformed lines, SKMEL2 and VMM39. The data demonstrate a wide diversity of responses between the cell lines to the combination of PLX4720 and lapatinib, regardless of whether the primary driving oncogene was BRAF or NRAS.

**Figure 6:**
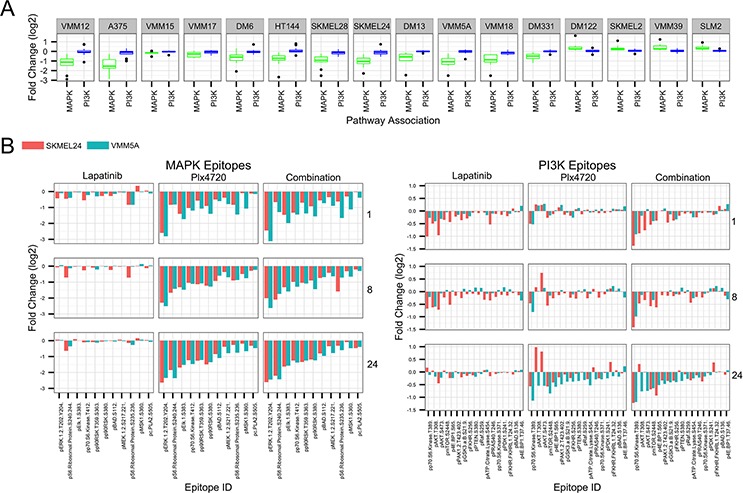
Changes in PI3 Kinase and MAP Kinase pathway protein phosphorylations in response to drug treatment **A.** Whisker plot of normalized, log2 transformed RPPA fold changes (PLX4720 treated over untreated) were plotted for MAPK pathway (green) and PI3K pathway (blue) epitopes for 12 BRAF mutant and 4 BRAF wt melanoma cell lines. Epitopes were selected from the arrays shown in Supplementary Figure S3 that were determined to be associated with the MAPK or PI3K signaling pathways, as shown in Panel B. Lines are ordered from most to least sensitive to PLX4720 treatment by IC50. **B.** Normalized, log2 transformed RPPA fold changes (drug treated over untreated) were plotted for SKMEL24 (red) and VMM5A (blue). Epitopes were selected from the arrays that were determined to be associated with the MAPK signaling pathway (left) or PI3K signaling pathway (right). Each facet of the plot represents the fold changes induced by treatment with lapatinib, PLX4720, or the combination (left to right) after 1, 8, or 24 hours (top to bottom).

The variety of adaptive responses can easily be visualized by focusing on a panel of MAP Kinase and PI3 Kinase pathway phosphorylation sites within the RPPA array (Figure 6). Figure 6A shows the inhibition of the MAP Kinase pathway by PLX4720 as a single agent in the BRAFV600E melanomas and their activation in the NRAS melanomas, based on the data displayed in Supplementary Figure S3. Blockade of BRAF in the BRAFV600E melanoma lines resulted in a drop in activating phosphorylations throughout the MAP Kinase pathway, including the transcription factor ELK1 and downstream kinases p90RSK and MSK1. Thus, pathway blockade in BRAFV600E melanomas was effective regardless of whether growth was inhibited. Lapatinib addition to PLX4720 did not significantly alter these phosphorylation changes, regardless of whether synergistic cytotoxicity was occurring (Supplementary Figure S3). Although PLX4720 as a single agent did not cause aggregate changes in PI3 Kinase pathway phosphorylations (Figure 6A), examination of the individual sites of phosphorylation (Figure 6B) revealed changes potentially informative of the mechanism of synergy with lapatinib in SKMEL24, but not in VMM5A. In the case of SKMEL24 (red bars), addition of lapatinib reversed the activating effect PLX4720 had on AKT phosphorylation, and deepened the inhibitory effects on the other phosphorylations, consistent with the hypothesis that lapatinib is synergistic in these cells by inhibiting PI3 Kinase signaling. By contrast, in VMM5A cells, addition of lapatinib to PLX4720 did not significantly alter any of the PI3 Kinase pathway phosphorylations beyond their response to PLX4720 alone, suggesting that the RTK inhibitor generated synergy by inhibiting a pathway other than MAP Kinase or PI3 Kinase in these cells. This is consistent with the empirical data of Figure 1, showing that the PI3 Kinase inhibitor PI103 had no synergistic benefit when combined with PLX4720 in these VMM5A cells.

**Figure 7:**
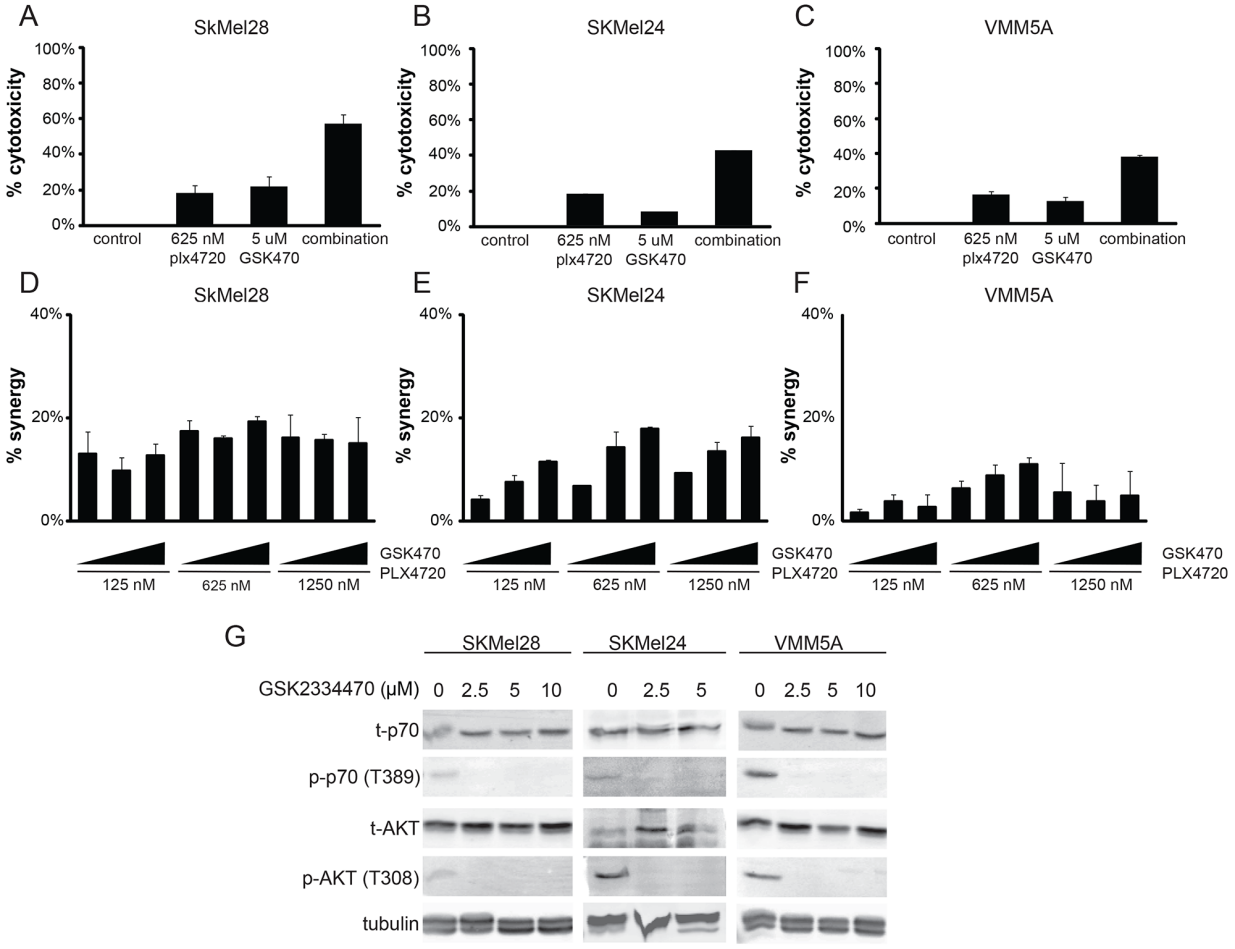
PDK1 inhibition can cause synergistic cytotoxicity in SKMEL24 and not VMM5A melanoma cells **A, B, C.** B-raf mutant cells SkMel28, SkMel24 or VMM5A were treated with vehicle control, 625 nM PLX4720, 5 μM GSK2233470, or the combination for 3 days. Metabolic activity was read out using alamarBlue (*n* = 3). **D, E, F.** Dose dependent synergistic benefit was determined in cells concurrently treated with PLX4720 (125 nM, 625 nM, or 1250 nM) and GSK2233470 (1.25 μM, 2.5 μM, or 5 μM) for 3 days. AlamarBlue was used to read out metabolic activity. Percent synergy is displayed for each dose combination (*n* ≥ 3). **G.** SkMel28, SkMel24, and VMM5A were treated with varying doses of GSK2233470 for 1 hour. Total protein was isolated and immuno-blotted for phospho (T389) and total p70S6K, phospho (T308) and total AKT, and tubulin.

Inspection of Figure 1 Panel C reveals that the putative PDK inhibitor, OSU-03012, provided the same pattern of synergies as the RTK inhibitors lapatinib and masitinib. This suggests that inhibition of this enzyme, a component of the PI3 Kinase signaling pathway, could be a key element in the adaptive resistance to PLX4720 in these cells. To test this concept, we examined the effects of GSK2334470, another PDK1 inhibitor with a different chemical structure. We found that GSK2334470 also displayed synergistic cytotoxicity with PLX4720 in SKMEL24 but to a lesser extent and over a narrow concentration range in VMM5A (Figure 7) consistent with the RPPA data which indicated that the PI3 Kinase pathway was not a major component of adaptive resistance in VMM5A. (We suspect that the broader effectiveness of OSU-03012 is due to a lack of specificity). These findings support the proposal that in at least some melanomas that utilize PI3 Kinase signaling as a survival mechanism, PDK1 can be an important component, as suggested by Ronai and colleagues [35, 99].

**Figure 8:**
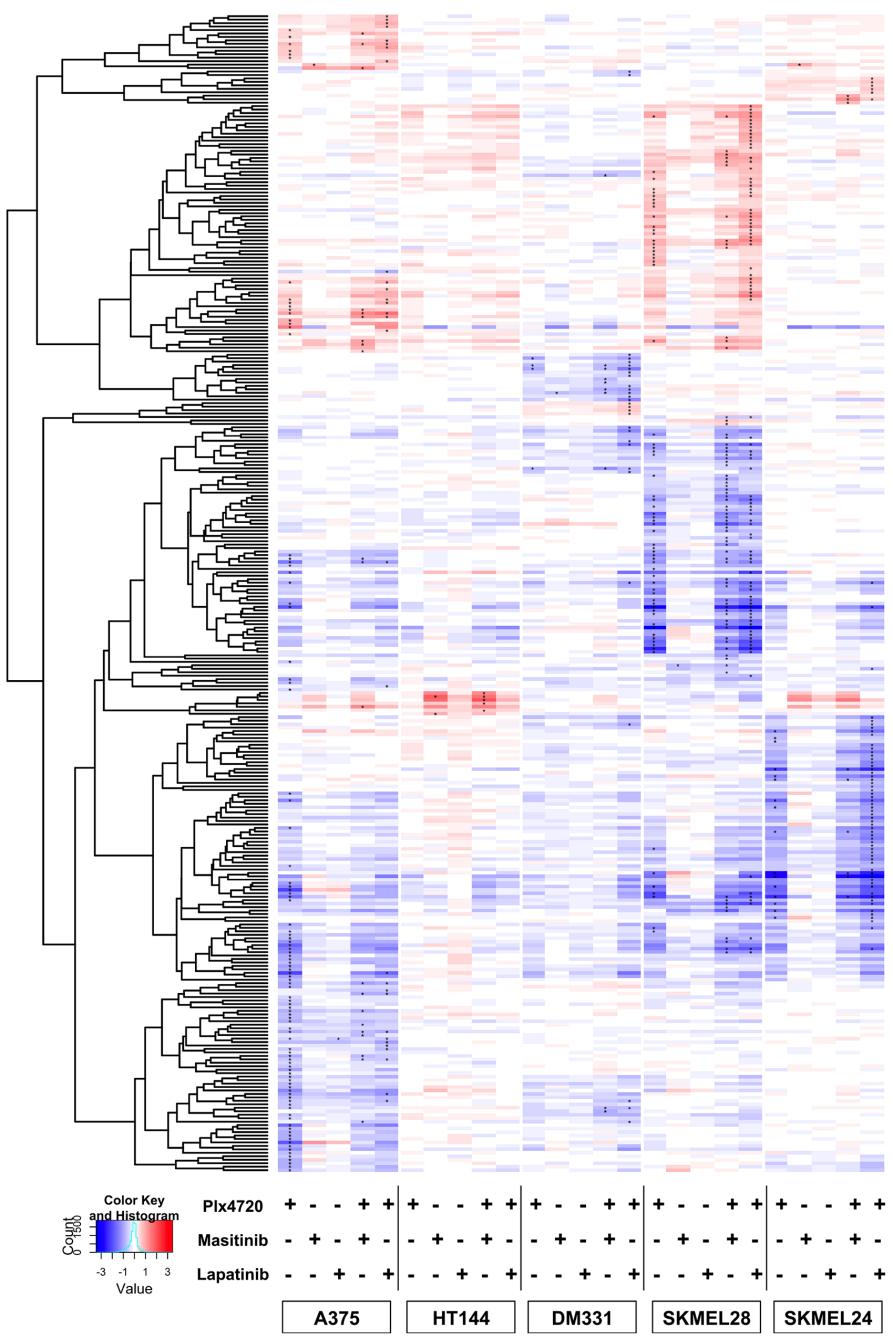
Heat Map of adaptive transcriptional changes in response to drug treatment Cells were treated for 8 hrs with either PLX4720, Masitinib, Lapatinib, the combination of Plx4720 and Masitinib, or the combination of Plx4720 and lapatinib. RNA was extracted as described in Methods. Using a moderated t-test, we identified differentially expressed genes after exposure to treatment in at least one sample. Unsupervised hierarchical clustering of log2 fold changes for genes, (y-axis) using correlation distance and average linkage. Genes with an FDR better than 1% are denoted with “*”.

To further broaden our analysis of drug-induced adaptive changes in cell signaling we profiled the transcriptional responses in five lines to treatment for 8 hours with PLX4720, lapatinib, masitinib or the combinations using Illumina HT12 v4 arrays (Figure 8). Inspection of the heat map of transcriptional changes shows broadly different responses to treatment with PLX4720 alone. A375 had a robust transcriptional response, which is expected given its strong sensitivity to the drug. The slightly resistant and non-synergistic line HT144 and the highly resistant line DM331 showed very modest transcriptional responses. However, SKMEL24 and SKMEL28, both of which are partially resistant to PLX4720 as a single agent showed very large drug-induced changes in transcription pattern. Importantly, each line displayed different blocks of genes responding to the various treatments. Each line's transcriptional response occupies an individual cluster, with few genes demonstrating significant responses across multiple lines.

Using MSigDB, a tool that aggregates multiple data bases, a pathway enrichment analysis was performed inputting differentially expressed genes in response to PLX4720 for the three lines that demonstrated synergy (SKMEL24, SKMEL28, and DM331). 91 unique gene sets were identified across the three sets of differentially expressed genes, but only two were shared by all three lines in response to PLX4720 alone: PID_AP1 and PID_NFAT_TF. The AP1 and NFAT pathways are stress response pathways that are nearly universal and were also seen in the sensitive A375 line, and thus are not viewed as signatures of cytotoxic synergy.

None of the lines showed large gene expression changes in response to addition of lapatinib or masitinib as single agents. Addition of the RTK inhibitors amplified some of the transcriptional effects of PLX4720 treatment, but what is most striking is that the majority of genes whose expression is altered by RTK inhibition only appear during combination treatment. Thus, it is clear that the BRAF inhibition was creating the vulnerability to the RTK inhibitor. Nevertheless there is considerable divergence in the gene expression changes induced by each RTK inhibitor and in each cell line. For example, of the 37 transcriptional changes induced by the PLX4720-lapatinib combination in DM331 cells, only 8 overlap with the 13 induced by the PLX4720-masitinib combination (Supplementary Figure S4 and Supplementary Table S2). No MSigDB gene sets were found to be enriched for all three lines post treatment with either combination. After treatment with the combination, DM331 was the only line that lost the enrichment for the PID_AP1_PATHWAY and PID_NFAT_TFPATHWAY.

Potential mechanistic insight could be gained by examination of individual gene lists that could be obscured by aggregating the genes into the pathways defined by standard databases. The lines displayed widely varying transcriptional indicators of combination treatment (Supplementary Table S2). DM331 responses centered on ERBB pathway ligands (TGF-alpha, epiregulin, neuregulin, HB-EGF) as well as other secreted growth factors (CSF-1, VEGFA). Thus, the regulatory bias by which DM331 presumably attains its resistance to BRAF inhibition (expression of EGFR and NRG-1) is enhanced in its adaptive response and inhibited at least in part by lapatinib. SKMEL24 displayed unique cytokine responses (IL-1, IRAK, IL-8, CXCL-1, CXCL-20) as well as genes associated with migration and extracellular matrix (Semaphorins, Serpin8, thrombospondin, and others). SKMEL28 combination responsive genes centered on ERBB pathway and cell cycle genes, including CDKN2A. Effects on MAP Kinase pathway-related genes also appeared in this list, notably the DUSPs (dual specificity phosphatases). Thus, although a logical hypothesis and list of potentially “actionable” targets could be constructed for each individual cell line about how growth inhibition and synergistic interactions might occur, no unifying themes emerged that could be applied to all the lines.

## DISCUSSION

### Combinatorial drug screening reveals diverse adaptive survival responses of BRAF melanoma cell lines

Because clinical responses to BRAF inhibitor treatment of BRAF mutant melanomas are generally incomplete, there is a compelling need to identify actionable mechanisms of intrinsic and rapidly appearing adaptive resistance to MAP Kinase pathway inhibition. To accomplish this, we screened a panel of 12 BRAF mutant melanoma cell lines for drugs or small molecule inhibitors that were synergistically cytotoxic with one of three MAP Kinase pathway inhibitors (PLX4720, RAF265 or PD325901). We found that no two cells were identical in their pattern of combinatorial drug sensitivities, although all were of the same developmental lineage, were driven by mutationally activated BRAF, and had elevated levels of MAP Kinase. This surprising result indicates that the broad genetic and/or epigenetic landscape of these cells, in addition to the primary oncogenic driver, determines the cell signaling architecture and hence the mechanisms of adaptation and resistance available to these cells. We refer to these functionally significant secondary genetic and epigenetic changes as “back-seat drivers.”

More synergies were observed in combination with RAF265 than with the other primary drugs. We suspect that this is because RAF265 is the least selective of the kinase inhibitors [100] and that some of the synergistic cytotoxicities are generated by “off-target” effects of RAF265. This raises the possibility that the most effective agents to use in combination therapy will be drugs that have multiple targets, a conclusion also reached by Langdon et al and Girotti et al. [63, 101]. The panel of drugs synergistic with the MEK inhibitor, PD325901, represents a subset of the drugs that enhanced cytotoxicity of the BRAF inhibitor, PLX4720. Assuming that PLX4720 effects are due solely to inhibition of RAF family members, this suggests additional activities of RAF beyond MAP Kinase activation, consistent with other reports [102–106]. Although the use of small molecule inhibitors in this analysis makes it difficult to draw unambiguous conclusions about molecular mechanisms, they potentially provide more clinical relevance than would putatively more specific reagents such as shRNA.

### Signaling by receptor tyrosine kinases represents a major source of adaptive resistance

Half of the cell lines tested, and all of the lines displaying some level of intrinsic resistance to BRAF inhibition, displayed enhanced cytotoxicity when PLX4720 was combined with lapatinib, consistent with the results of Held et al. [56]. The same lines also responded to the combination of PLX4720 with masitinib, an inhibitor of PDGFR, KIT and VEGFR tyrosine kinases, but not of HER family kinases. These results suggest that reduction in the total RTK activity rather than inhibition of a specific RTK may be important for enhancing the effectiveness of PLX4720. Consistent with this concept, we found that gefitinib, which is selective for EGFR and does not inhibit other HER family kinases, did not achieve the 98^th^ percentile synergy threshold obtained with lapatinib although, as reported by Girotti, Marais and colleagues [71, 72], some additive benefit was obtained in some cell lines. Ng et al have reported that the pan-HER family inhibitor canertinib provides even greater benefit [107] and Langdon et al. have recently reported that dovitinib,a multi-tyrosine kinase inhibitor, displays synergistic benefit comparable to the combination of 6 separate kinase inhibitors [63]. These observations all support the proposal that feedback-regulated activation of RTKs is a central component of adaptive resistance in melanomas, and that drug combinations that focus on this causal agent have clinical potential.

RTK inhibitors had modest effects on gene expression when used as single agents, but much larger effects when combined with the BRAF inhibitor. This indicates that, even though there is enormous diversity in patterns of gene expression and of changes in protein phosphorylation (as measured by RPPA) across individual lines, upregulation of RTK dependency is a convergent feature of the adaptive response to PLX4720 in many BRAF mutant melanomas. Consistent with this, group-wise analysis of gene expression of 4 resistant cell lines treated as replicates (SKMEL28, VMM5A, VMM18, DM13; excluding DM331 because of its high basal EGFR expression) by Pathway Express showed upregulation of genes associated with the ERBB pathway following PLX4720 treatment [61]. Notably, group-wise analysis allows modest but consistent changes to be identified across a group of cell lines while they cannot be identified by analyzing each cell line separately. The upregulation of ERBB family members generates the vulnerability to lapatinib inhibition in these lines.

### Multiple adaptive survival pathways contribute to RTK-dependent resistance to BRAF inhibition

We did not find that differences in the IC50 for pERK inhibition nor the extent of MAP Kinase pathway reactivation correlated with combination drug sensitivity (Figures 4 and 5, Supplementary Figures 2 and 3) indicating that resistance to PLX4720 via RTK activation does not occur solely by reactivation of the MAP Kinase pathway. Rather, our data indicate that resistance to PLX4720 via RTK activation depends also on engagement of other pathways known to be accessible to RTKs, such as PI3K and JAK/STAT. Although data from patients shows that therapeutic response correlates with MAP Kinase pathway inhibition by vemurafenib [5, 108, 109] we suggest that inhibition of MAP Kinase signaling by RAF inhibitors is a necessary but not a sufficient condition to achieve cytotoxicity.

It is widely recognized that a major mechanism of resistance to MAP Kinase inhibition in cancer is generated via the PI3 Kinase pathway. For example, Akt mutations can play a role in acquired resistance to vemurafenib, and Akt inhibitors and PI3 Kinase inhibitors can be synergistic with BRAF inhibition [21, 24, 29, 37, 38, 56, 110–117]. In agreement with this, we observed that the PI3 Kinase inhibitor PI-103 was synergistic with PLX4720 in some of the resistant cell lines. OSU-03012, a putative PDK1 inhibitor, displayed synergistic benefit with PLX4720 in all of the cell lines that were resistant or partially resistant to the BRAFV600E inhibitor. Ronai and colleagues have also pointed to an important role of PDK1 in melanoma [99]. We therefore examined the effects of GSK2334470 which was developed as a specific PDK1 inhibitor, and found that it also provided synergistic cytotoxicity with PLX4720 in SKMEL24, a cell line where lapatinib altered the profile of selected PI3 Kinase pathway phosphorylations. Interestingly, the PDK1 inhibitor was less effective at generating synergy in VMM5A, a cell line where lapatinib addition to PLX4720 generated synergy without apparent effects on PI3 Kinase signaling. These data demonstrate that PLX4720-lapatinib synergy can be achieved independent of effects on PI3 Kinase signaling as well as MAP Kinase reactivation.

The fact that some cell lines sensitive to PDK1 inhibition were not sensitive to PI3 Kinase inhibition indicates that even though PDK1 is “downstream” of PI3 Kinase, inhibition of these targets is not equivalent. This agrees with the surprising results recently reported by Scortegagna et. al. showing synergy between PI3K and PDK1 inhibitors [35, 36] in BRAF mutant melanomas.

Collectively, our data demonstrate that there is enormous diversity in the adaptive survival responses to BRAF inhibition utilized by different BRAFV600E melanomas, and that many of the changes observed by molecular profiling are therapeutically irrelevant. Thus, it will be extremely challenging to develop drug combinations that specifically inhibit each significant adaptive response. Our data suggest that drug combinations that pair MAP Kinase pathway inhibition with other individual intracellular signaling pathways, such as PI3K, STAT or WNT, may not be as broadly useful as combinations with an RTK inhibitor that dampens multiple pathways. Thus we suggest that a combination of MAP Kinase pathway inhibition with the addition of an RTK inhibitor such as lapatinib is worthy of clinical evaluation.

## MATERIALS AND METHODS

### Cell culture, antibodies, and reagents

SkMel28 cells (American Type Culture Collection; ATCC; Rockville, MD), A375, DM13, DM331, DM6, HT144, SKMel24, VMM12, VMM15, VMM17, VMM18, and VMM5A (kind gift from Dr. Craig Slingluff, University of Virginia) were propagated in RPMI Medium 1640 (Invitrogen, Grand Island, NY) supplemented with 5% or 10% fetal bovine serum (FBS; Gemini Bio-Products, West Sacramento, CA). All cultures were maintained in a humidified chamber at 37°C with 5% CO_2_. OncoMap analysis was conducted at the Broad Institute to identify the mutational status of over 30 known oncogenes and tumor suppressor genes. The cell lines were authenticated by comparing the tumor mutation profile determined by OncoMap with published reports.

Antibodies were obtained from the following sources: anti-phosphoERK and anti-total ERK (Sigma-Aldrich), and anti-tubulin (Calbiochem, Gibbstown, NJ).

Sources of combination inhibitors are shown in Supplementary Table S1. PLX4720 was a gift from Plexxikon, RAF265 a gift from Novartis and PD325901 a gift from Pfizer.

### Synthetic lethal pathway screen

Combinatorial drug screening was as described previously [62]. In brief, prior to screening, plate density that resulted in 80% confluence after three days of incubation was determined for each cell line. We observed batch-to-batch variability in lots of fetal calf serum that altered the ability for synergistic interactions to be manifest, so each lot was tested at 1.0% to 5% serum. A dose response of each inhibitor used in the screen was performed on a panel of cell lines to determine doses that would result in approximately 15, 25, and 35% growth inhibition. Cell lines were grown in their normal growth media to 80% confluence and then were washed with 1x PBS, trypsinized, collected, counted (via hemocytometer), and resuspended in phenol-red free RPMI 1640 + FBS at concentrations that would result in 80–90% confluence of the vehicle-treated control wells after 3 days of growth. Plating of the cells was carried out using the BioMek NX workstation (Beckman Coulter, Indianapolis, IN). 90 μl of cell suspension was added per well in 96-well format. Small molecular inhibitors were diluted to 10x concentration and plated into master drug plates. The BioMek NX workstation was used to add 10 μl of drug from the master plates to each well. The cells were then incubated for 3 days at 37°C and 5% CO2. Following this incubation, the BioMek NX workstation was used to add 10 μl of alamarBlue (Invitrogen, Grand Island, NY) to each well. The plates were incubated for 4 hours and fluorescence was measured at 560 nm excitation/590 nm emission on a Synergy 2 plate reader (BioTek Instruments, Winooski, VT). Mean results and standard error were calculated for triplicate samples.

The Bliss model of independence was used to determine synergy [76, 77]. This model estimates the combined effect of two drugs as the multiplicative effect of each drug measured individually: Synergy Score = CCombination − (1 – (1 – CSecondary Drug)*(1 – CPrimary Drug)) where CCombination is the observed cytotoxicity of the combination treatment, CSecondary Drug is the observed cytotoxicity of the secondary drug, and CPrimary Drug is the observed cytotoxicity of the primary drug. Bliss independence assumes that the two drugs act through independent mechanisms, and is thought to be a reasonable baseline model for large networks [77]. When the observed experimental data match the predictions of Bliss independence, the inhibitors are said to be additive whereas greater than predicted potency indicates synergism and lower potency indicates antagonism. An advantage of this method is that it can score synergy even when one of the drugs is inactive as a single agent.

### Animal studies

All experiments were carried out under an Institutional Animal Care and Use Committee (IACUC) approved protocol and institutional guidelines for the proper and humane use of the animals in the research were followed. Four- to six-week-old female Hsd:Athymic Nude-Foxn1nu mice were obtained from Harlan Laboratories (Indianapolis, IN) and maintained in ventilated caging. Tumors were generated by injecting 1 × 10^7^ cells in 1:1 Phosphate-Buffered Saline: Matrigel (BD Biosciences, San Jose, California) subcutaneously into the dorsal flanks (2 sites per mouse). Mice were anesthetized by intraperitoneal injection of 3 ml/kg of a solution containing 25 mg/ml ketamine HCl, 2.5 mg/ml xylazine, and 14% ethanol in 0.85% NaCl. Surgical sites were prepped and cells were delivered with a 25 gauge syringe in 100μl volumes. Tumors were allowed to grow to an average volume of 400 mm^3^ ((length * width^2^)π/6) and mice were randomized into groups for treatment. Mice received vehicle control and/or control rodent diet, 200 mg/kg lapatinib (LC Laboratories) alone by oral gavage once daily, rodent diet with 417 mg/kg plx4720 (Research Diets, Inc. New Brunswick NJ), or plx4720 rodent diet plus 200 mg/kg lapatinib by gavage. Tumors were measured bi-weekly and mice were euthanized by cervical dislocation under anesthesia when tumors reached 1000 mm^3^. A random coefficient model was used to estimate mean tumor growth following treatment initiation, assuming a quadratic model and with a robust estimate of the covariance matrix [118]. F-tests based on the random coefficient models were used to compare the groups. Analyses were carried out in SAS 9.4, PROC MIXED.

### Immunoblot analysis

Cells were allowed to adhere to plates overnight before being treated with inhibitors or vehicle control in phenol red-free RPMI Medium 1640 (Invitrogen, Grand Island, NY) without fetal bovine serum for 1, 8, or 24 hours at 37°C. Cells were rinsed with cold 1x PBS containing phosphatase inhibitors, calyculin A and orthovanadate, and lysed in Triton lysis buffer [10% Triton X-100, 5% 1M Tris (pH 7.5), 2.5% 4M NaCl, 0.5% 0.5M NaF, 0.01% 0.5M EDTA, 80% water] plus the following protease and phosphatase inhibitors: 1 μg/ml pepstatin, 1 μg/ml leupeptin, 0.4 TIU/ml aprotinin, 1 mM phenylmethylsulfonyl fluoride, 200 μM orthovanadate, 50 mM Δ-glycerophosphate, and 0.4 μM Microcystin. Immunoblot analysis was as described (Roller, 2012)

### Reverse-phase protein arrays

RPPAs were performed as previously described [119]. Briefly, cell lysates were diluted with 2 × Tris-Glycine SDS Sample Buffer (Life Technologies Corporation, Carlsbad, CA, USA) before printing on nitrocellulose slides (Grace Bio-Labs, Bend, OR, USA) and were spotted in triplicate with the Aushon 2470 contact pin arrayer (AushonBioSystems Inc., Billerica, MA, USA), in 4-point twofold dilution curves.

### Gene expression microarrays

Cells were collected and RNA was isolated using the Qiashredder (Qiagen) and RNeasy Mini Kit (Qiagen). RNA was quantified on the NanoDrop 2000 spectrophotometer (Thermo Scientific) and RNA quality was inspected on a 1% agarose gel. Biotin labeled RNA was hybridized to Illumina 3′IVT human HT-12 BeadChip arrays.

### Statistical analysis of functional genomics and genomics data

Illumina microarray data was variance stabilized transformed [120, 121] using the *lumi* Bioconductor package in R [122]. Differentially expressed genes were identified using *limma* to perform moderated *t*-tests and derive Benjamini-Hochberg False Discovery Rate (FDR) adjusted *p*-values [123, 124] and applying a 1% FDR threshold. Differentially expressed genes were clustered using the R package *pvclust* [125] with the Pearson correlation distance measure and average linkage. We assessed the significance of the clusters by performing 1000 iterations of the clustering introducing random variations and assessing how much randomness was required to lose a specific branch. Normalized log2 reverse phase protein array (RPPA) data was generated using methods described in [121]. We performed a paired *t*-test in limma to identify epitopes which were affected by treatment using a 1% FDR.

All gene expression files will be available from the GEO database (http://www.ncbi.nlm.nih.gov/geo/), accession number: GSE68453. All exome files available from Dryad (doi:10.5061/dryad.9gn07). Detailed analysis is in Capaldo et al [61].

## SUPPLEMENTARY FIGURES AND TABLES






