# Supplementary material for: Combinatorial drug screening and molecular profiling reveal diverse mechanisms of intrinsic and adaptive resistance to BRAF inhibition in V600E BRAF mutant melanomas
Source: Oncotarget. 2015 Dec 10;7(3):2734–53. doi: 10.18632/oncotarget.6548 (PMC4823068; doi:10.18632/oncotarget.6548)
Supplement: Supplementary file 1 [file oncotarget-07-2734-s001.pdf]

SUPPLEMENTARY FIGURES AND TABLES

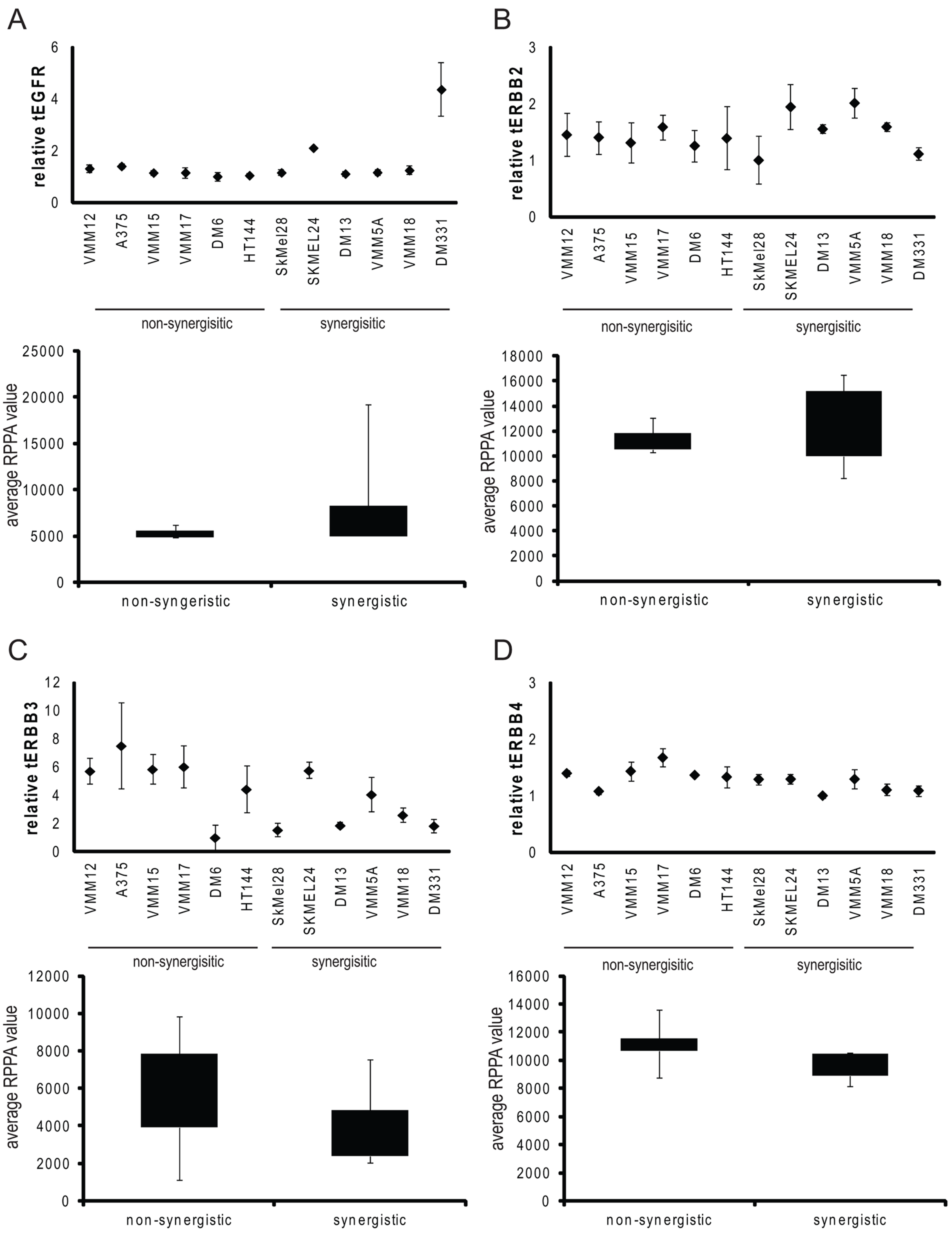

Supplementary Figure S1: HER Family expression levels in melanoma cell line panel. Control cell lysates were analyzed by Reverse Phase Protein Arrays for the presence of ERBB family receptors. Average RPPA value and the standard error of the mean for the synergistic cell lines and the non-synergistic cells as calculated for **A.** tEGFR **B.** ERBB2.

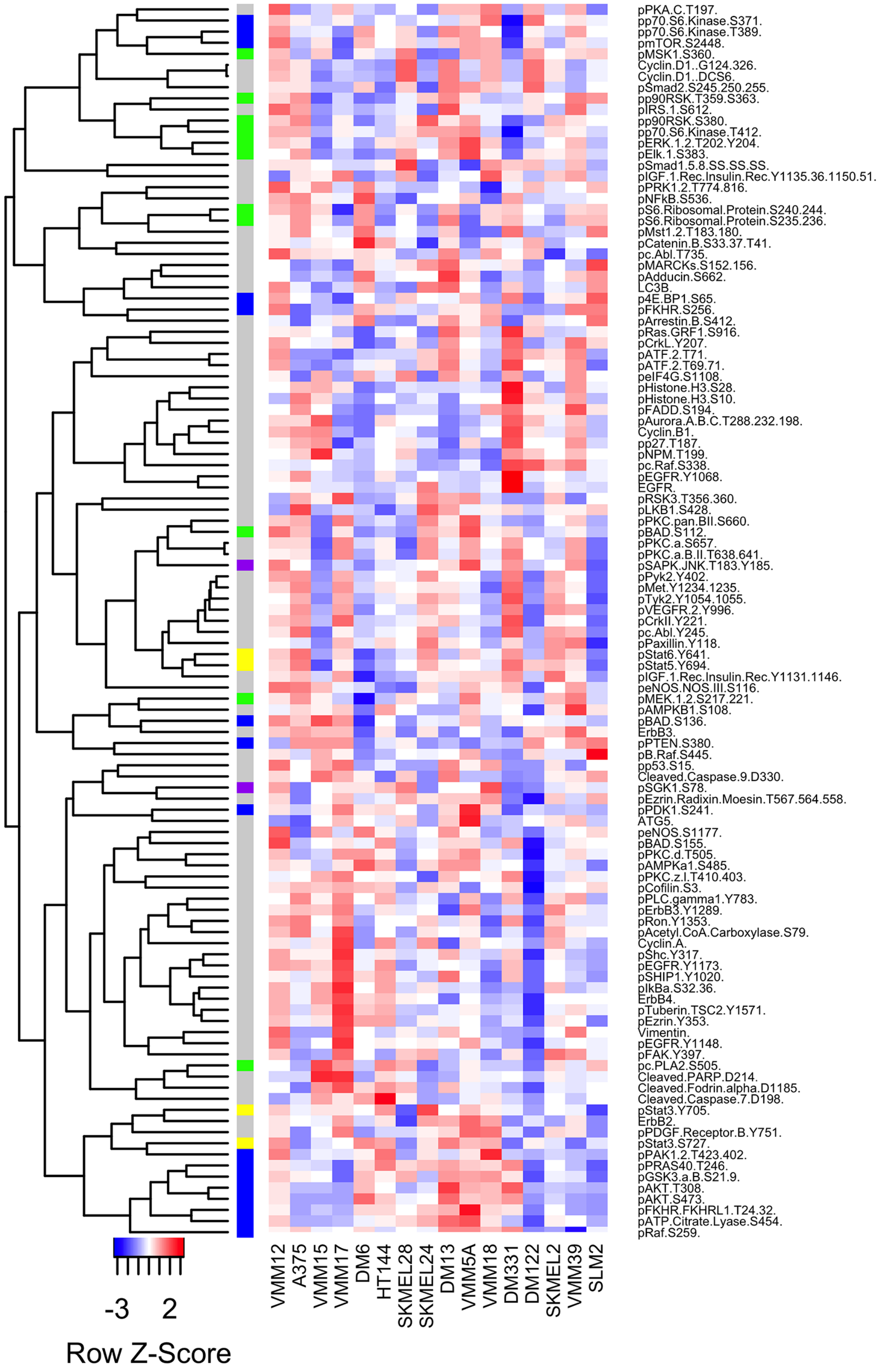

Supplementary Figure S2: Heat map of one way ANOVA analysis of basal phospho-epitopes determined by RPPA. Using a one way ANOVA test, we selected for epitopes that varied between cell lines at a FDR of 1% or better. Unsupervised hierarchical clustering of row scaled epitopes' intensities (y-axis) using correlation distance and average linkage is shown. Cell lines were ordered by sensitivity to PLX4720. Unsupervised clustering using correlation distance and average linkage of their epitopes' intensities did not order cell lines in proportion to drug sensitivity or driver oncogene. Pathway membership is denoted along the y-axis by the presence of colored boxes for the MAPK pathway (Green), PI3K pathway (Blue), stress (Purple), or STAT signaling (Yellow).

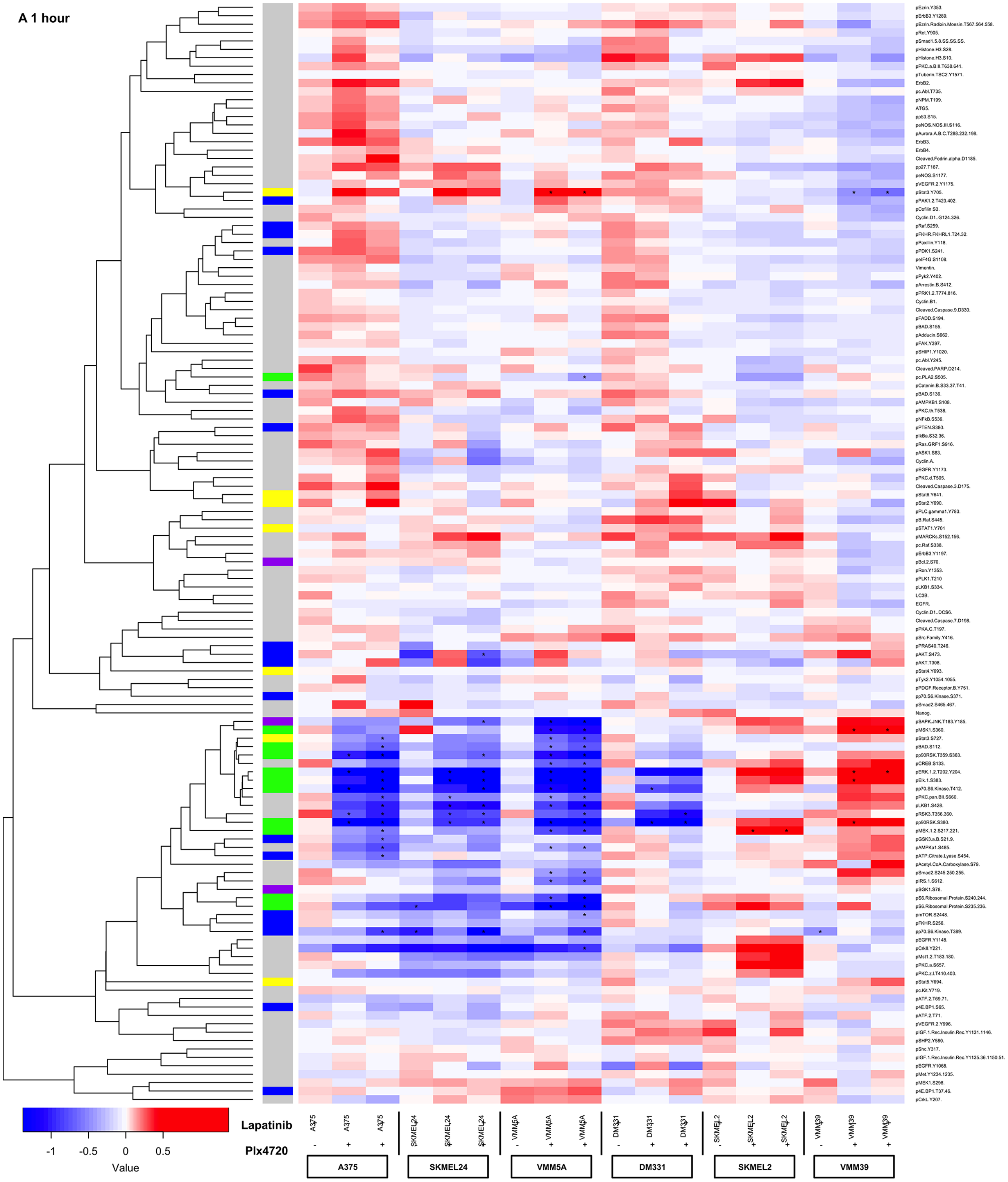

Supplementary Figure S3: Heat Maps of RPPA analysis of melanoma cell lines treated with PLX4720 with and without lapatinib, at 1, 8 and 24 hrs. (*Continued*)

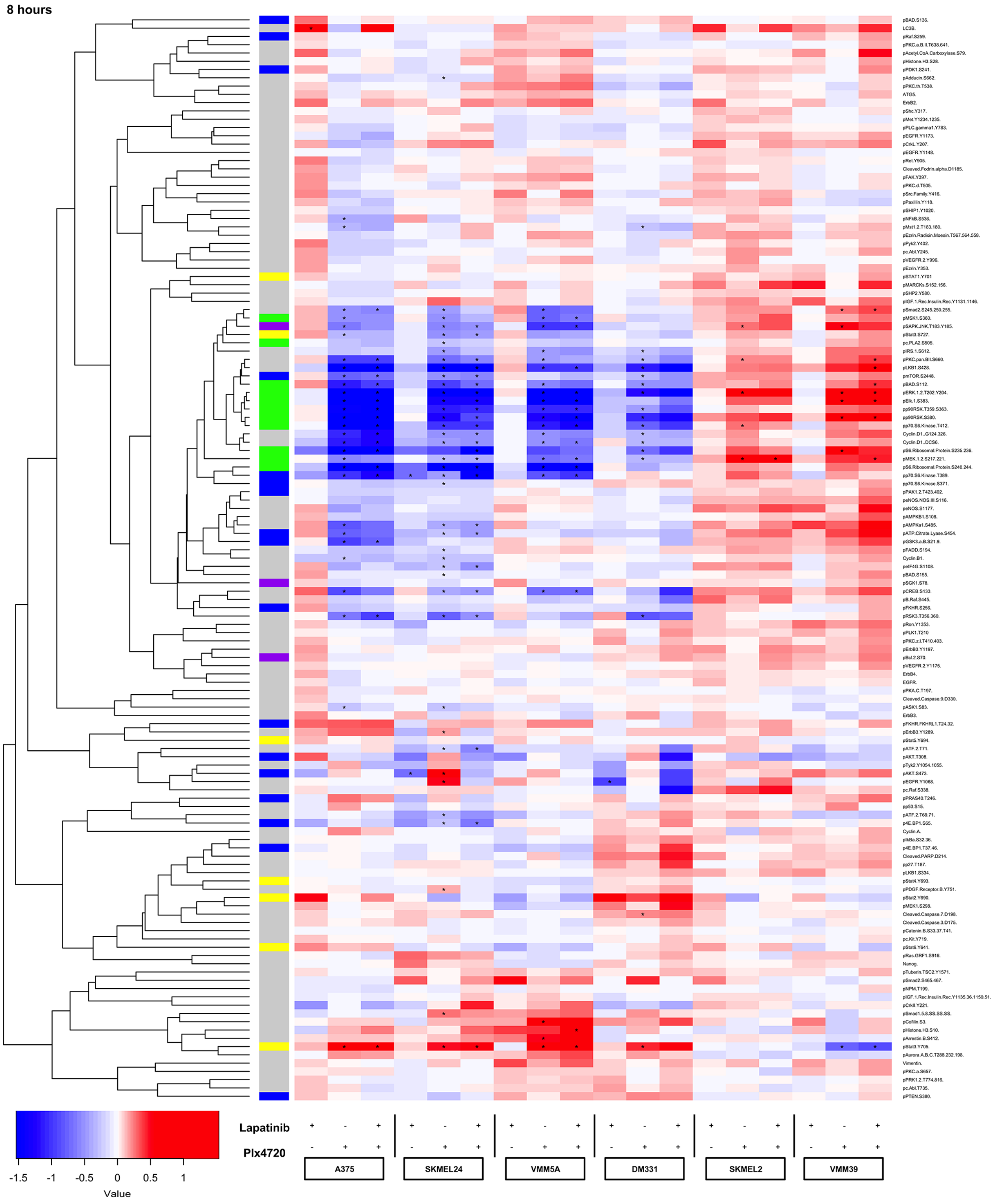

Supplementary Figure S3: (*Continued*) Heat Maps of RPPA analysis of melanoma cell lines treated with PLX4720 with and without lapatinib, at 1, 8 and 24 hrs. (*Continued*)

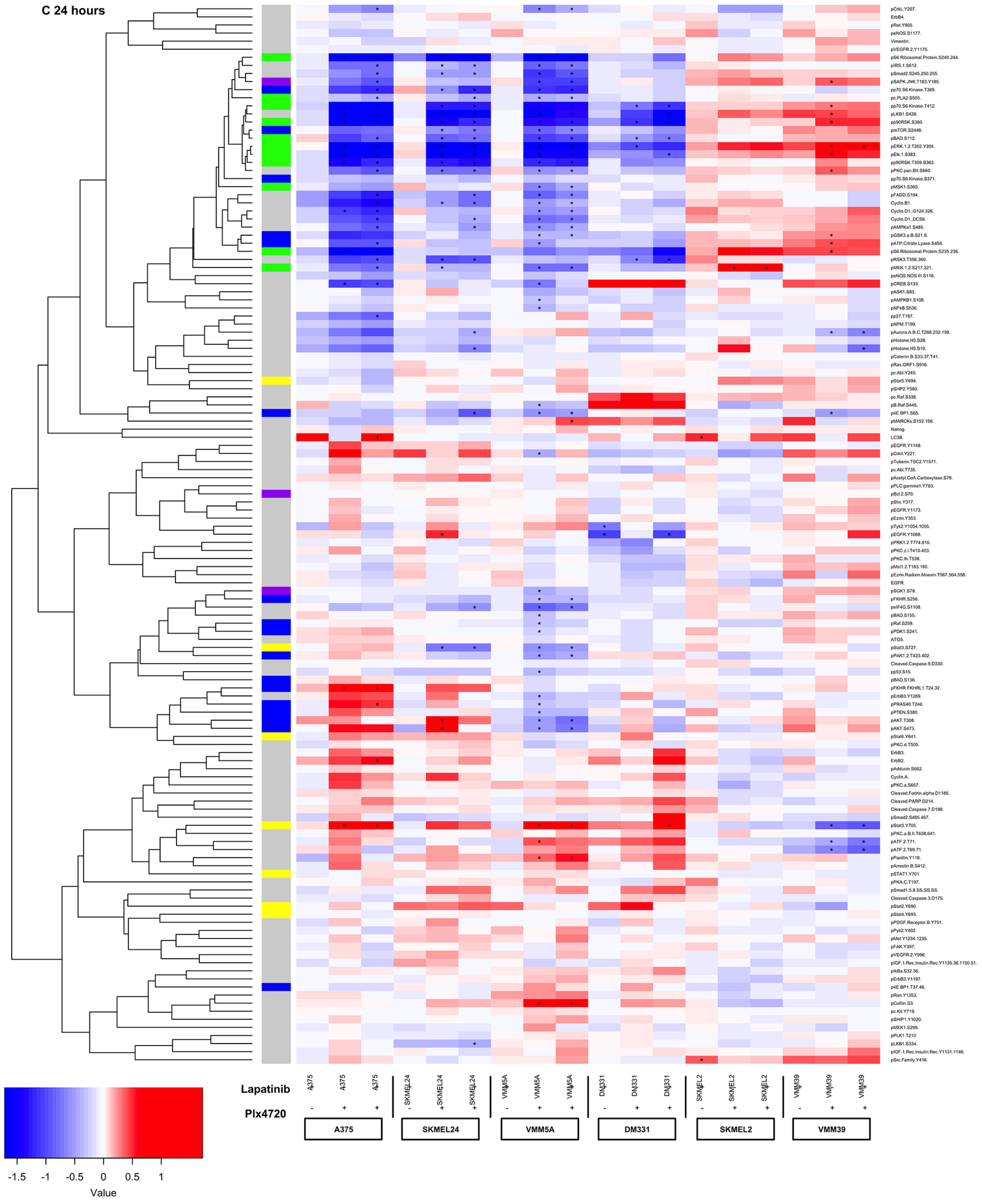

Supplementary Figure S3: (*Continued*) Heat Maps of RPPA analysis of melanoma cell lines treated with PLX4720 with and without lapatinib, at 1, 8 and 24 hrs. 4 BRAFV600E and 2 BRAFWT melanoma cell lines were treated for 1, 8 or 24 hours with 125 µM PLX 4720 with or without 4 µM lapatinib. Cells were lysed as described and analyzed by Reverse Phase Protein Arrays. Differentially abundant or differentially phosphorylated epitopes were identified using a moderated *t*-test, and epitopes with an FDR of better than 1% are marked with an asterisk ("*"). Unsupervised hierarchical clustering of log2 fold changes for epitopes (y-axis) was performed using correlation distance and average linkage. Cell lines are ordered by sensitivity to PLX4720 (IC50). Pathway membership of epitopes is denoted along the y-axis by the presence of colored boxes for the MAPK pathway (Green), STAT (yellow), PI3K pathway (Blue), stress pathways (Purple).

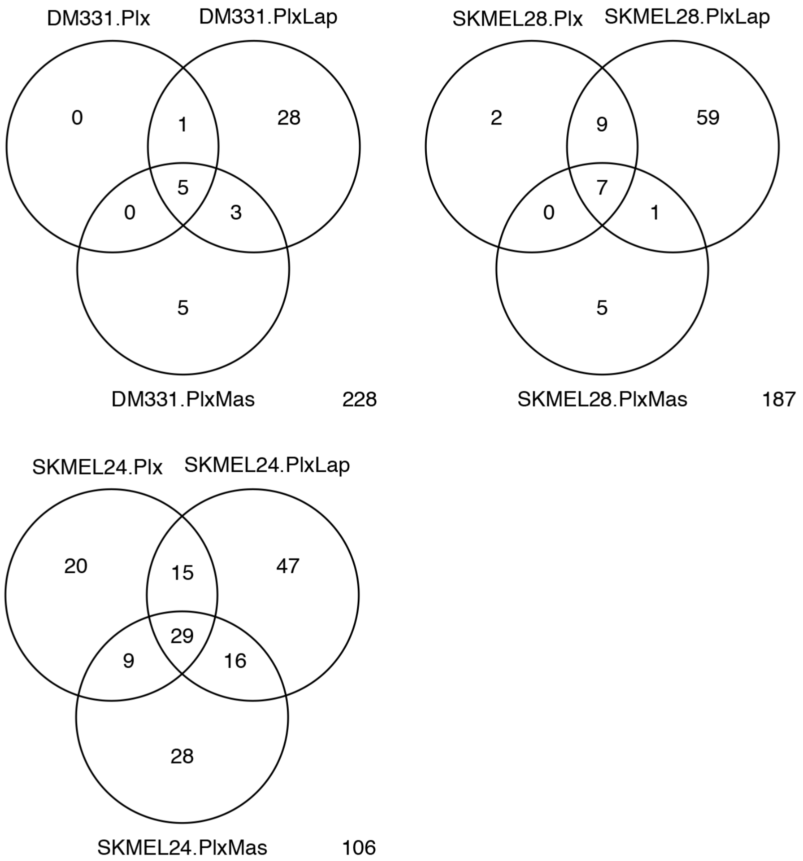

Supplementary Figure S4: Venn diagrams of genes induced by PLX4720 with or without addition of lapatinib or masitinib.

Supplementary Table S1: Inhibitors used in the cytotoxicity screen

Supplementary Table S2: Genes induced by PLX4720, lapatinib, masitinib or the combinations

Table of annotated probes from Illumina HT12 v4 gene expression microarray that were differentially expressed at a FDR of 1% or better after treatment with Plx4720 alone (PvC), lapatinib alone (LvC), masitinib alone (MvC), Plx4720 combined with lapatinib (PLvC), or Plx4720 combined with masitinib (PMvC), for cell lines DM331, SKMEL24, and SKMEL28. Differential expression log2 fold changes were calculated using Bioconductor package limma and a moderated ***t***-test was used to assess significance.
